# Cognitive state monitoring and the design of adaptive instruction in digital environments: lessons learned from cognitive workload assessment using a passive brain-computer interface approach

**DOI:** 10.3389/fnins.2014.00385

**Published:** 2014-12-09

**Authors:** Peter Gerjets, Carina Walter, Wolfgang Rosenstiel, Martin Bogdan, Thorsten O. Zander

**Affiliations:** ^1^Hypermedia Lab, Knowledge Media Research CenterTübingen, Germany; ^2^Department of Computer Engineering, University of TübingenTübingen, Germany; ^3^Department of Computer Engineering, University of LeipzigLeipzig, Germany; ^4^Team PhyPA, Biological Psychology and Neuroergonomics, Technical University BerlinBerlin, Germany

**Keywords:** passive brain-computer interface, EEG, cross-task classification, working-memory load, adaptive learning environments, cognitive load theory

## Abstract

According to Cognitive Load Theory (CLT), one of the crucial factors for successful learning is the type and amount of working-memory load (WML) learners experience while studying instructional materials. Optimal learning conditions are characterized by providing challenges for learners without inducing cognitive over- or underload. Thus, presenting instruction in a way that WML is constantly held within an optimal range with regard to learners' working-memory capacity might be a good method to provide these optimal conditions. The current paper elaborates how digital learning environments, which achieve this goal can be developed by combining approaches from Cognitive Psychology, Neuroscience, and Computer Science. One of the biggest obstacles that needs to be overcome is the lack of an unobtrusive method of continuously assessing learners' WML in real-time. We propose to solve this problem by applying passive Brain-Computer Interface (BCI) approaches to realistic learning scenarios in digital environments. In this paper we discuss the methodological and theoretical prospects and pitfalls of this approach based on results from the literature and from our own research. We present a strategy on how several inherent challenges of applying BCIs to WML and learning can be met by refining the psychological constructs behind WML, by exploring their neural signatures, by using these insights for sophisticated task designs, and by optimizing algorithms for analyzing electroencephalography (EEG) data. Based on this strategy we applied machine-learning algorithms for cross-task classifications of different levels of WML to tasks that involve studying realistic instructional materials. We obtained very promising results that yield several recommendations for future work.

## Introduction: passive brain-computer interfaces for adaptive digital environments

A Brain-Computer Interface (BCI) is a direct link between a human brain and a technical system. It detects patterns in brain activity (by means of so-called classifiers) and translates them into input commands for the machine. Usually, brain activity is recorded noninvasively through electroencephalography (EEG) and is interpreted by a conventional personal computer using machine learning and signal processing techniques (Blankertz et al., 2002). Machine learning algorithms are used to classify different patterns in the EEG that have to be actively produced by patients, e.g., by imaging movements (Wolpaw et al., 1991; Pfurtscheller et al., 1993; Schalk et al., 2004) or that are elicited in reaction to an attended stimulus. The initial, principal goal of BCI-based applications has been to provide direct communication and control channels for patients who have lost their ability to communicate naturally (Wolpaw et al., 2002). As the reliability and usability of BCI systems have improved over the past decade, their applicability and their appeal for other purposes besides patient support has grown also. Today BCI-approaches are developed for applications beyond assistive technologies addressing general problems of human-computer interaction (Zander et al., 2010). According to such a passive BCI approach (Zander and Kothe, 2011), spontaneously generated brain signals related to current changes in cognitive and affective user states can be deployed to support a given interaction. With passive BCIs, new methods for real-time cognitive state assessments become available that might improve human-computer interaction significantly (for exemplary studies in this emerging field see e.g., Lin et al., 2005; Ramsey et al., 2006; Dyson et al., 2010).

Different forms of passive BCIs have been proposed in recent years, for example for detecting the perception of errors (Zander and Jatzev, 2009; Zander et al., 2010), for detecting cheating (Reissland and Zander, 2010), for detecting specific intentions when fixating an object (Zander et al., 2011a; Protzak et al., 2013), or for detecting the perceived loss of control when using a system (Jatzev et al., 2008; Zander and Jatzev, 2012). Passive BCIs can be considered as an implicit, secondary communication channel enriching the ongoing primary human-computer interaction by providing information about ongoing user states. The opportunity to adapt specialized digital environments to current mental states such as inattention, cognitive or perceptual workload, mental fatigue or aversive emotions might be very helpful for users such as system operators, people interacting in augmented environments, or even surgeons and astronauts (Tonin et al., 2010; De Negueruela et al., 2011).

In this paper we will mainly concentrate on issues related to the detection of users' cognitive workload and the automatic adaptation to it. Workload adaptation is a topic that is not only relevant for specialized digital environments as mentioned before but also for much more ubiquitous and generic settings of everyday life such as adaptive learning environments for educational purposes. As will be outlined in the next section, adapting the complexity of instructional materials to learners' cognitive workload has been the main rationale of many instructional design approaches. In the remainder of this paper we will elaborate on the methodological and theoretical prospects and pitfalls of a passive BCI approach to cognitive workload assessment in instructional contexts by discussing results from the literature and from our own research. Based on these discussions, we will present a strategy on how several inherent challenges of applying BCIs to working-memory load (WML) and learning can be met by refining the psychological constructs behind WML, by exploring their neural signatures, by using these insights for sophisticated task designs, and by optimizing algorithms for analyzing EEG data.

## Cognitive workload and adaptive instruction: a challenge for instructional design

According to important cognitive theories of instructional design (e.g., Cognitive Load Theory, CLT, Sweller et al., 1998; Cognitive Theory of Multimedia Learning, CTML, Mayer, 2009) the type and amount of cognitive load learners experience while studying instructional materials is one of the crucial factors for successful learning. Optimal learning conditions are characterized by providing challenges for learners without inducing cognitive overload. This general rationale is also prevalent in many classic instructional design theories. For instance, Vygotsky's (1978) well-known “zone of proximal development” describes an ideal instructional situation as intermediate between situations that are “too difficult” and situations that are “too easy.” Salomon's (1984) AIME-approach focuses on optimizing learners' “amount of invested mental effort” by manipulating the perceived task demands of learning materials in a way that learners' are stimulated to engage in study activities imposing a high level of cognitive workload. Reigeluth's seminal Elaboration Theory (Reigeluth and Stein, 1983) is based on the idea of presenting the same instructional contents in a sequence of increasingly more complex versions to align the cognitive workload imposed onto learners to their growing level of understanding. Thus, presenting instructional materials in a way that learners' cognitive workload is constantly held within an optimal range is not a new idea but rather seems to be unequivocally advocated by many instructional theories as a guideline to provide optimal learning conditions. The different conceptions, however, of what constitutes the nature of cognitive workload, seem to be less unequivocally.

Workload theories in the human factors area like Wickens' (1984) widely adopted Multiple Resource Model assume that there are numerous information-processing resources (like perception, processing and action applied to items of different modalities and representational codes), each of which could potentially be overloaded in a current task performance, thereby generating a bottleneck. On the contrary, cognitive theories of instructional design like CLT or CTML are much more specific with regard to the information-processing resources they consider as pivotal cognitive bottlenecks for processes of learning and comprehension. According to these theories, the workload imposed onto a structure called working memory is the crucial type of cognitive load. Working memory describes the small amount of information that can be held and manipulated in mind simultaneously for the execution of a current cognitive task (Cowan, 2014). A careful management of cognitive load imposed onto working memory is the main instructional rationale of theories like CLT or CTML. This is in line with the main recommendation derived by working memory researchers (e.g., Cowan, 2014) from their work with regard to improving learning and education: It is of fundamental importance that instructional materials are adjusted to the working-memory capabilities of learners.

The concept of WML in CLT and CTML refers back to the multicomponent working memory model by Baddeley (1986, 2000, 2012), which distinguishes verbal and visual temporal storage components (phonological loop and visuospatial sketchpad) from an attentional control system (the central executive, borrowed from the supervisory attentional system postulated by Norman and Shallice, 1986). CLT and CTML focus almost entirely on the capacity limitations of the storage components of working memory as the bottlenecks for learning, assuming that these components constrain the amount of new information that can be processed simultaneously in order to be integrated into long-term memory. According to this view, if the amount of information a learner has to process at some point in time, exceeds the capacity of these storage components, the entire learning process is hindered. Consequently, the scientific literature on CLT and CTML focuses on identifying instructional manipulations that allow influencing the type and amount of WML during learning in order to not overtax students working memory capacity.

WML during learning is commonly assumed to be the result of an interaction between the external task requirements of the instructional design presented to learners and the complexities of the contents to be learned in relation to learners' knowledge prerequisites (cf. Kalyuga et al., 2003). Identical contents can result in lower WML for more (as compared to less) knowledgeable learners due to the chunking of information enabled by the availability of more complex concepts for representing contents. Beyond the cognitive load imposed by the representational holding of contents (also described as intrinsic or essential cognitive load) there are additional components of cognitive load imposed by task requirements of the instructional design itself (e.g., requirements such as searching and handling information, comparing instructional materials, or drawing elaborative inferences). Depending on whether these additional processes required by the instructional design are helpful or hindering for deeper learning they are described as germane or extraneous processes (Sweller et al., 1998; Gerjets and Scheiter, 2003; Gerjets and Hesse, 2004; Cierniak et al., 2009b). This distinction, however, between “positive” (germane) and “negative” (extraneous) components of cognitive load has been subject to severe critique in recent years (cf. Gerjets et al., 2009; De Jong, 2010).

Frameworks like CLT and CTML have elaborated ample advice on how to adapt contents and instructional designs to students' limited working-memory resources. One important caveat in this field, however, consists in the fact that it is not possible to provide a general advice for all types of learners independent of their level of prior knowledge. Due to the interaction between content complexities and learner's knowledge prerequisites, one and the same instructional material can impose different levels of workload onto different learners. There are even “expertise reversal effects” showing that one instructional material *A* as compared to another instructional material *B* imposes higher WML for novice learners, whereas the reverse is true for more advanced learners (Kalyuga et al., 2003). For instance, a so-called integrated graphic that presents pictorial elements in close spatial proximity to corresponding verbal explanations (e.g., labels) imposes a lower level of WML onto novice learners than a non-integrated graphic that provides verbal explanations apart from their pictorial counterparts (e.g., as a figure caption). It is assumed that integrated graphics support novices in searching for verbal explanations of pictorial elements. Interestingly, the inverse pattern has been shown for more advanced learners (e.g., Cierniak et al., 2009a). Advanced learners suffer from higher WML when learning with integrated graphics as compared to non-integrated graphics. This is due to the fact that at some point during learning verbal explanations of pictorial elements become redundant for learners as they are acquainted with these elements. Subsequently, learners might tend to even suffer from integrated graphics with regard to WML because they are forced to process verbal information in working memory that is unnecessary for them to understand the graphics. Consequently, from the viewpoint of instructional-design theories WML is a highly volatile learner characteristic, potentially consisting of different cognitive load components that change during learning not only due to learners' increasing level of knowledge but also due to the changing instructional materials and task requirements presented to learners at each point in time.

Accordingly, an ultimate instructional goal would probably be a moment-to-moment assessment of WML leading to an immediate online adaptation of instructional materials in case of learners getting overwhelmed (or underchallenged) with regard to their currently available working-memory capacity. A natural technological solution to achieve this goal would consist in constructing an adaptive learning environment that—other than traditional intelligent tutor systems (ITS) based on model-tracing (e.g., Corbett, 2001) or based on natural language analysis (e.g., Graesser and McNamara, 2010)—does not aim to diagnose learners' developing knowledge structures directly, but more generically concentrates on the continuous detection of different levels of WML and the online-adaptation to it. Probably, the biggest obstacle to achieve such a technological goal is the lack of appropriate measurement procedures allowing for a continuous and non-obtrusive online tracking of WML. Current measurement methods mostly rely either on subjective workload ratings (Hart and Staveland, 1988; Paas et al., 2003) or on dual-task procedures (Brünken et al., 2003; DeLeeuw and Mayer, 2008; Cierniak et al., 2009b), both of which are likely to disrupt and annoy learners while studying—without allowing for a fine-grained temporal modeling of WML. Despite these drawbacks, first provisional attempts to construct workload-adaptive learning environments based on subjective rating scales yielded promising results, even though adaptation was not instantaneous, as WML could not be assessed continuously (Mihalca et al., 2011). Additionally, the learning process had of course to be interrupted frequently in this study to obtain workload ratings from learners.

Less obtrusive measures of WML that are better integrated into the learning process, such as performance measures obtained on assessment items embedded into the instructional materials are unfortunately not sufficiently specific for cognitive load monitoring, as they do not reveal whether the successful or unsuccessful solution of an assessment item was achieved with a high or low level of working-memory investment. Or as Cowan (2014) puts it: “One potential pitfall to watch for is that, while some students will want to press slightly beyond their zone of comfort and will learn well, others will want an easy time, and may choose to learn less than they would be capable of learning,” Thus, the investments of working-memory resources during learning and the success or failure with regard to assessments of learning outcomes are clearly dissociable. Beyond this disadvantage, even repeated assessments during learning will not lead to a continuous measurement and might even frustrate learners in case of high failure rates.

In sum, measures like task performance, rating scales, or dual tasks do not allow for a continuous and non-obtrusive measurement of WML and this fact provides a fundamental problem for workload-adaptive instructional environments. One option that has been advocated to overcome this problem is to identify suitable physiological indicators of WML. For instance, it has been proposed to use physiological measures like pupil dilation or skin conductivity for a continuous WML assessment. However, up to now these measures mostly turned out to be not very reliable and specific indicators of WML (Brünken et al., 2003; Paas et al., 2003). A novel methodological avenue for solving this problem in instructional research might be to obtain more direct physiological measures of neural activity to derive sufficiently specific indicators of WML during learning. This approach can capitalize on a tradition of using EEG parameters as workload indicators in human factors research as well as in neuroscience research (e.g., Gevins and Smith, 2003). In instructional scenarios this approach has been applied very rarely and in a tentative fashion (e.g., Gerlic and Jausovec, 1999; Antonenko and Niederhauser, 2010; Antonenko et al., 2010). In this paper we will follow this line of research by trying to explore the potential of a passive BCI approach for designing workload-adaptive learning technologies based on EEG signals. In the near future, such BCIs may be applied in combination with low cost headset-like EEG sensors using dry electrodes (as they are currently beginning to reach the market, cf., Zander et al., 2011b) to help learners in instructional environments to keep their cognitive workload constantly within an optimal range. Beyond preventing cognitive overload, such BCIs may also help to immediately detect and fix a lack of challenge when learning materials are too simple in relation to a specific learner's prerequisites in terms of prior knowledge or working memory capacity. This approach resembles ITS that have already been developed for other types of learner states (e.g., affective states like boredom or engagement), following the same rationale of using sensor-based measures to control adaptive learning environments (for an overview see Calvo and D'Mello, 2010).

## Promises and drawbacks of EEG-based measurement of WML during learning: the issue of perceptual-motor confounds

The currently best-studied EEG correlates of WML that would be suitable for a continuous online assessment of learner states are variations in the oscillatory power of the theta and alpha band activity (for a review see Klimesch, 1999). An increase in WML has repeatedly been shown to lead to an increase in theta band activity over frontal-midline electrodes (event-related synchronization, theta ERS; e.g., Gevins et al., 1997; Jensen and Tesche, 2002; Missonnier et al., 2006; Krause et al., 2010; Sauseng et al., 2010) and a decrease in alpha band power over parietal-occipital electrodes (event-related desynchronization, alpha ERD; e.g., Gevins et al., 1997; Stipacek et al., 2003; Krause et al., 2010).

In line with these findings, it has recently been proposed by Antonenko et al. (2010) to use oscillatory power for the measurement of WML in instructional research. To substantiate this idea, the authors discuss two studies that obtained continuous EEG measures to indicate WML during learning with hypermedia (Antonenko and Niederhauser, 2010) and multimedia (Gerlic and Jausovec, 1999). However, in our view both of these studies are subject to one of the main pitfalls that have to be resolved before neural workload measures can find a useful application in the context of a passive BCI to improve learning: In both studies it remains very unclear due to the complex learning materials used for experimentation whether the observed EEG differences between more and less demanding learning materials really go back to differences in WML alone or whether they might be mostly results of some behavioral correlates of workload or of some other perceptual or motor differences between the experimental conditions.

For instance, Gerlic and Jausovec (1999) found that learning about planets from spoken text combined with music and pictorial information (high WML) as compared to learning from written text alone (low WML) yielded alpha ERDs in temporal and occipital electrodes whereas in the text only condition alpha ERDs in frontal and central electrodes occurred. Here, potential differences in WML between the experimental conditions are massively confounded with perceptual differences of the experimental materials. The same criticism applies to the second study cited by Antonenko et al. (2010). This study (Antonenko and Niederhauser, 2010) revealed differences in alpha and theta frequencies when reading hypertext with vs. without link previews, hypothetically indicating a lower level of WML in conditions with link previews. However, conditions with link previews differed also perceptually (pop-up windows showed up) and with regard to motor activity (mouseover was needed for activating previews) from the conditions without previews. Again, these perceptual-motor confounds seem to prevent a clear attribution of the EEG differences found between experimental conditions to levels of WML. In our view, these problems of perceptual-motor confounds seem inevitable when using standard EEG power analyses for comparing different realistic learning materials (instead of comparing more controlled experimental tasks without perceptual-motor confounds).

What implications do these studies have with regard to the prospects of developing workload-adaptive instructional environments based on a passive-BCI approach? First it has to be noted that the standard EEG power analyses used in these experimental studies require a statistically averaging of data across several subjects and trials to identify significant EEG differences between instructional conditions. When it comes to adaptive learning environments, however, single-subject single-trial analyses are necessary to derive a continuous classification of learners' level of WML from EEG data allowing instantaneous reactions to different levels of WML. This requires very different approaches to online data analysis. Second, even if it is possible to classify realistic instructional conditions (inducing different levels of WML) online based on suitable methods to analyze EEG power differences, the question needs to be addressed, whether this classification is really based on differences in WML or on some of the perceptual-motor confounds of the different instructional conditions. In the latter case, it can unfortunately not be expected that the classification method used for the current task will yield transfer to novel instructional situations unless these situations have similar perceptual-motor confounds of different levels of WML. Beyond this problem that a classification based on perceptual-motor confounds is quite uninteresting from a practical perspective, it is also quite uninformative with regard to the theoretical issue of whether we can adapt instructional environments to cognitive workload because the classification method might not be specific enough to address workload only and hence might not really keep track of learners WML. We addressed both caveats (demonstration of single-subject single-trial analyses; avoiding perceptual-motor confounds in classifier training) in two of our own studies that will be reviewed in this paper.

## Study 1: realistic instructional materials imposing different levels of WML: do single-subject single-trial EEG data allow for a classification of these levels?

The first caveat that we addressed in a study by Walter et al. (2011) is whether EEG power differences between learners studying two types of realistic instructional materials (inducing different levels of WML) are sufficiently strong and reliable to enable a good classification result when analyzed with BCI methods. The problem of analyzing single-subject single-trial data online is pivotal for the passive BCI approach and requires completely different methods for analyzing oscillatory EEG data than those used by Antonenko and Niederhauser (2010). These methods are based on machine learning algorithms as they have been developed by research on traditional BCIs for patients. In our study, we used realistic materials similar to Gerlic and Jausovec (1999) or Antonenko and Niederhauser (2010) to investigate classification accuracies, being aware, of course, that using realistic instructional materials will inevitably result in the abovementioned confound issues.

The study by Walter et al. (2011) used a within-subject design to manipulate WML. Ten learners (12–14 years) were asked to study two different types of instructional materials involving processes of learning and comprehension at different levels of WML. In a high WML study window, learners had to study graphical representations and explanations of mathematical angle theorems in order to understand the theorems. In a low WML study window, learners had to study a different kind of graphical representations, namely comic strips, in order to understand the stories depicted. Both materials involved complex graphical displays, however, the comic strips where quite easy to understand for learners whereas the angle theorems where quite hard for them to grasp. The two types of materials where presented to learners in an alternating sequence to avoid confounding WML with presentation time. This type of sequencing was chosen to improve experimental control and internal validity. It has to be noted, however, that it is not very representative with regard to realistic instructional situations. Under realistic conditions, there is usually an increase in the objective complexity of learning materials over time, which is, however, not always associated with an increase of learners' level of WML due to learner's improved knowledge prerequisites over time (see Section Cognitive workload and adaptive instruction: A challenge for instructional design for details).

The experimental procedure comprised three parts. First, subjects had to solve a pre-test to assure they had no prior knowledge on angular geometry before participating in the study. A second part consisted of three learning episodes, 11 min each. In each episode subjects were asked to study five angle-theorems as well as five comic strips (in alternating sequence). Each theorem and each comic strip was presented for a 45 s time interval that we define as *study window*. Subsequent to each study window, subjects were requested to answer a question with regard to the interpretation of the theorem or comic strip (four multiple-choice options presented for 10 s). Finally, subjects had to rate their subjective level of cognitive load during the study window on a 7-point Likert scale presented for another 10 s. Overall, subjects were presented with 15 study windows on angle theorems and 15 study windows on comic strips in the second part of the experiment, always alternating between these two types of materials. In a third part of the experiment, first, learning outcome measures were obtained by asking students to solve angle problems. We used a German version of the Carnegie Learning geometry tutor for this task (Schwonke et al., 2007). Finally, a post-test comprising the same items as the pretest was administered in order to allow for direct pre-post comparisons.

During the two types of study windows (angle theorems vs. comic strips), EEG data was collected and features with regard to different characteristics in the EEG frequency bands were extracted. Due to the low number of 30 study windows (15 for each type of instructional material), all artifact free study windows were segmented into smaller epochs of 15 s for each subject and each study window. Accordingly, 45 epochs per subject resulted for each type of instructional material (3 epochs per study window × 5 study windows per learning episode × 3 learning episodes for each type of instructional material), which were used for classification. This segmentation was possible because no significant variation was detected in the signal over a full study window. Concerning the feature selection, we focused on frontal and parietal electrodes with regard to the spectral power within the alpha (8–13 Hz) and theta (4–7 Hz) frequency band. For spectral analysis, an autoregressive model was calculated using the Burg-Algorithm. As there were no theta differences between learning materials, only alpha power values were used as features to classify comic epochs (studying easy materials inducing low levels of WML) vs. theorem epochs (studying difficult materials inducing high levels of WML). As students were not required to take any overt motor action during learning, prima facie no obvious motor confounds of the two types of study windows are to be expected. The results show that during epochs with high levels of WML, a desynchronization of alpha band activity could be observed in parietal (and occipital) brain areas as compared to epochs with low levels of WML. These differences could be successfully classified on a single-subject single-trial basis by using a support vector machine (SVM) with radial basis function (RBF)-Kernel (Lotte et al., 2007). A 10-fold cross validation was conducted to verify the accuracy of the trained SVM. The mean classification accuracy for all 10 learners was 76%. For seven out of the ten learners, an accuracy of 80% or higher could be achieved for detecting epochs with high vs. low levels of WML.

Thus, our results show that it is indeed possible to classify whether learners study realistic instructional materials inducing low vs. high levels of WML with a substantial accuracy based on single-subject single-trial EEG data. However, although our results seem to be practically relevant and methodologically interesting, we would consider them to be subject to the same conceptual critique that we raised when discussing the studies by Gerlic and Jausovec (1999) or Antonenko and Niederhauser (2010) with regard to perceptual-motor confounds. For instance, the angle theorems and the comic strips might differ with regard to certain perceptual characteristics, potentially leading to differences in processing beyond imposing different levels of workload onto working memory (e.g., different eye-movements or different types of semantic processing). Thus, even if there have been no obvious motor confounds of task difficulty in this study (because students were not required to take any overt motor action during learning), there nevertheless might have been perceptual and as a consequence subtle motor confounds because more difficult tasks might have resulted in different eye-movements that could have been picked up by a classifier. Accordingly, our second caveat remains: Even if an EEG-based classification is possible for realistic tasks imposing different levels of WML, this classification might still not be very helpful theoretically because it remains unclear whether a workload classifier trained on realistic tasks really represents a measure of WML or of something else.

## Are there successful classifications of levels of WML based on oscillatory EEG data? a critical reflection on recent studies and some suggestions for improvement

Having a closer look at the tasks used for the classification of WML from EEG data in studies outside the field of learning and instruction reveals that problems of perceptual-motor confounds seem to be quite common, shading doubt onto the usefulness of these results as examples for the successful classification of WML. Interestingly, these problems even occur in most studies that use simple and low-level working-memory tasks for classification.

For instance, Heger et al. (2010) compared a resting state situation to a situation where subjects were conducting workload-imposing tasks like flanker tasks (where they have to press keys in reaction to the orientation of the middle arrow of a display of five arrows like: > > < > >) or switching tasks (where they have to press keys to decide whether a number is greater vs. lower than five or whether a number is odd vs. even depending on the dashed or solid framing of the number). Heger et al. (2010) classified the neural signature of the resting state situation vs. the workload-imposing situations with an accuracy of over 90% [using Artificial Neural Networks (ANNs)]. Additionally, they applied the classifier trained on these manipulations to realistic computer-based tasks of low vs. high WML (reading vs. typing) by using a so-called cross-task classification approach (for details with regard to this approach see below). The authors claim that the classifier application to the computer-based tasks was quite successful, although no quantitative results of this cross-task classification are reported. These results seem to be quite impressive at first sight, however, there are severe perceptual-motor confounds in this study that render a clear theoretical interpretation of the findings impossible: Obviously, a striking difference between the two classes used for training and for cross-task classification was whether motor activity was involved or not (key pressing vs. resting and typing vs. reading). Thus, motor activity was strongly confounded with the manipulation of WML. As the representation of motor activity in the EEG itself has a strong alpha component and there are strong oscillatory electromyographic effects (EMG) on the beta and gamma band activity in addition (which were actually used for classification by the authors) this study may mainly demonstrate a classification of motor vs. no-motor activity rather than of high vs. low levels of WML.

A study by Berka et al. (2004) who claim to classify different levels of workload in motor tasks as well as in cognitive tasks might be subject to a more sophisticated motor confound. They used EEG data to distinguish four classes of “vigilance” and report that increasing workload leads to a classification into the high vigilance class for all types of tasks. However, as their classifier mainly relies on alpha band power (which is also subject to ERD during motor activity) in combination with behavioral measures (fast eye blinks), it remains quite unclear whether the classifier is sensitive for increased workload, increased motor activity and eye movements, or both.

Similar arguments apply to the work of Chaouachi et al. (2011) who used digit span tasks (requiring subjects to retain sequences of digits of different length) and a logic task (requiring subjects to induce rules of different difficulties describing sequences of numbers like 2 - 4 - 6) for training and classification (by means of Gaussian Process Regression). The NASA-TLX rating scale (Hart and Staveland, 1988) was used to obtain subjective data on experienced cognitive workload. Their results yielded over 90% accuracy with regard to the prediction which tasks were subjectively rated as simple, intermediate or difficult. Though initially impressive, again the difficulty levels of the tasks used were systematically confounded with the amount of motor activities required. Thus, the results cannot unambiguously be interpreted as a classification of WML. For instance in the easiest condition of the digit span tasks subjects were prompted 20 times to enter the last three digits presented resulting in a pattern of 20 bursts of three key presses in the experimental block. In the most difficult condition of the digit span task they were prompted four times to enter the last eight digits presented resulting in a pattern of four bursts of eight key presses in the experimental block. Thus, not only does the easy block contain 60 key presses and the difficult block 32 key presses but also the temporal distribution was very different. Moreover, a baseline measurement that was used as a standard for the EEG power data from the different experimental blocks was obtained only once at the beginning of the experiment. In combination with the fact that all tasks were presented in an easy to difficult order, the study seems also to confound task difficulty and time delay since baseline measurement, which might be exploited by the classifier. Therefore, systematic drifts of EEG features over time could also be responsible for good classification results.

Another study that reported good within-task classification results for three types of working-memory tasks (two levels of WML each) is the study by Baldwin and Penaranda (2012). They used a reading span task, a visuospatial n-back task, and a Sternberg task for inducing different levels of WML. The reading span task requires simultaneous processing (deciding whether a sentence is correct or not) and storage (memorizing a letter presented at the end of each sentence) for a variable number of sentences (three or four in the low WML condition and six or seven in the high WML condition). The visuospatial n-back task required remembering and comparing the previous locations of a moving square with its current location. In the low WML condition the current location has to be compared with the location one trial before (1-back). In the high WML condition it has to be compared with the location three trials before (3-back). Thus, the task requires updating a list of one vs. three locations in working memory for each trial. Nine different locations were used for presentation and the probability that the current location was identical to the criterion location presented *n* trials before (i.e., positive match) was set to 50%. Subjects received a feedback (answer correct or not) after each trial. The Sternberg task requires deciding whether a number was or was not present in a list of numbers presented before. In the low WML condition the list length was one, two, or three. In the high WML condition the list length was four, five, or six. The six blocks of tasks (two difficulty levels of each of the three tasks) were presented in a randomized order. Each experimental block was presented for 5 min to subjects. For the subsequent EEG analysis each block was segmented into 60 non-overlapping windows of 5 s each (motor responses required to complete the tasks were not excluced from the analysis). The classifiers used to distinguish the levels of WML relied on 50 features, namely the EEG power of five frequency bands (delta, theta, alpha, beta, and gamma) obtained in 10 EEG channels (three frontal, three central, three parietal, and one occipital). ANNs were applied to classify the two levels of WML for all three tasks. The within-task classification, using 50% of the data from the to-be-classified task as training data and 50% for classifier application, resulted in high classification accuracies (approximately 80% on average). As in the studies reported before, however, these results might easily go back to some perceptual-motor confounds as the different experimental conditions (levels of WML) differed strongly with regard to the amount of motor activity contained in the EEG signal used for classification. For instance, in the reading span task the memorized letters had to be typed into an on-screen keyboard every three or four letters in the low WML condition and every seven or eight letters in the high WML condition. Thus, in the easy condition the number of 5-s windows that contain motor signals from typing should approximately be twice as large as in the difficult condition. The same is true for the Sternberg task. In the easy condition, a motor reaction is required after a sequence of one to three numbers, whereas in the difficult condition, a motor reaction is only required after a sequence of four to six numbers. Again, windows containing motor signals should be twice as frequent in the easy condition than in the difficult condition. The effects of these confounds might be severe as some of the EEG frequency bands used for classification (e.g., alpha or gamma) strongly react to motor responses. The n-back task used by the authors seems to have no obvious motor confounds with regard to the number of reactions required, however, the task might suffer from a subtle perceptual confound: The 1-back is a quite easy task with an error rate of approximately 10% whereas the 3-back is a rather difficult task with an error rate of approximately 30% (chance level was set to 50%). As subjects received a feedback (response correct or not) after each trial, the number of 5-s windows containing the cognitive processing of error feedback was three times higher in the difficult condition than in the easy condition. Assuming that error feedback leads to strong neural responses in the EEG (Falkenstein et al., 1991; Spüler et al., 2012a) it cannot be ruled out that this perceptual confound due to feedback presentation has contributed to the strong classification results. Moreover, as the visuospatial n-back requires subjects to memorize one vs. three locations on the screen, it cannot be ruled out that subjects in the difficult condition displayed much more eye-movements during rehearsal than subjects in the easy condition. As the authors do not report to have removed artifacts due to eye movements from the EEG data, this difference might also have contributed to good classification results.

Based on the abovementioned studies, it has to be noted that even if there are no obvious motoric confounds of task difficulty in workload classification studies—as it is, for instance, the case in the n-back task used by Baldwin and Penaranda (2012) or in our own study reported above (Walter et al., 2011)—it is nevertheless possible that perceptual confounds are present as more difficult tasks might result in different visual displays (e.g., more error feedback or more complex graphics) leading to differences in processing that are unrelated to the concept of WML (e.g., eye movements or error detection). Thus, in order to reliably classify WML, which is a precondition for our goal of developing workload-adaptive instructional environments, it is important to train EEG classifiers on tasks that are highly controlled for perceptual and motoric confounds, which probably cannot be fulfilled by the realistic instructional tasks that we eventually intend to classify in instructional environments.

With regard to the issue of avoiding perceptual-motor confounds of WML, the most convincing classification study we found in the literature was conducted by Brouwer et al. (2012), who used a sophisticated approach to workload classification based on a letter n-back task. They implemented three levels of WML (0-back, 1-back, and 2-back), whereby in the easiest condition (0-back) no updating was required as participants only had to compare whether a presented letter was identical to a previously defined target letter. Tasks were presented in blocks consisting of 48 trials of the same level of difficulty (2.5 s per trial, 2 min per block). Overall, 24 blocks were presented in a pseudorandom order (eight blocks of each difficulty level). The first six blocks of each difficulty level were used for classifier training, the remaining two blocks for classifier testing. Similar to the n-back task used by Baldwin and Penaranda (2012) no obvious perceptual-motor confounds were committed. Additionally, compared to the visuospatial n-back task used by Baldwin and Penaranda (2012) there were probably no differences between difficulty levels with regard to eye-movements for the letter n-back task. Moreover, Brouwer et al. (2012) controlled for potential effects of the differential distributions of error feedback received by participants for the three difficulty levels. In order to run the EEG analysis, each block was segmented into non-overlapping windows of different length depending on the analysis (ranging from 2.5 to 120 s per window, which allows to test for the relation between temporal window size and classification accuracy). Motor responses required to complete the tasks and other artifacts like eye blinks were not excluced from the analysis. Different types of classifiers were tested with regard to their ability to detect levels of WML. The classifier that was based on oscillarory power relied on three frequency bands (theta, alpha, and beta) obtained in seven EEG channels (frontal, central, and parietal). A SVM was applied to these features to classify different levels of WML. The results showed that the classification accuracy strongly depended on the temporal window size used for the classification (ranging from 2.5 to 120 s) as well as on the difficulty levels that have to be distinguished. The best classification results (approximately 85% correct on average) were obtained for distinguishing complete blocks of 0-back and 2-back task (i.e., decision after 48 trials or 120 s). However, as the 0-back task involves no updating process at all but relies a comparison of a current stimulus with a predefined target, it might not really be considered a working-memory task (as compared to the 1-back or 2-back task). Distinguishing the latter two (thereby involving a serious detection of the level of WML) yielded classification results of approximately 75% on average (again based on a window size of 48 trials or 120 s). However, the classification accuracy rapidly diminished when the time interval after which the decision had to be made decreased. For instance, for the distinction between 0-back und 2-back tasks the accuracy level was reduced from approximately 85% on average for a window size of 120 s (i.e., one block) to approximately 65% for a window size of 2.5 s (i.e., one trial). Unfortunately, the authors do not report on the accuracy of the more interesting distinction between 1-back and 2-back task for small window sizes but based on the abovementioned results it can be expected to be rather small. Thus, the overall classification results in this study seem to be sound and encouraging—but they are not overwhelming with regard to the prospects of yielding a fast real-time classification of WML as it is needed for developing workload-adaptive instructional environments. We assume that one reason for the moderate classification accuracy for short windows of analysis might be the fact that the motor responses required to complete the tasks as well as other artifacts like eye blinks were not excluced from the analysis in the study of Brouwer et al. (2012). As these artifacts are known to cause strong EEG signals, they can be expected to increase the overall noise level of the data, thereby potentially drowning out the weaker signals resulting from the different levels of WML implemented in the study. Accordingly, it might be a better option to define windows of analysis in a way that a time interval from at least approximately 125 ms before any keypress to approximately 125 ms after any keypress is excluded from the analysis to filter out the strongest motor signals for the sake of data quality. This is probably most important during classifier training. Using such a procedure during classifier application would be anyway quite difficult to implement in a realistic online scenario.

To conclude, several studies have recently been conducted to classify different levels of WML based on oscillatory EEG data. Although many authors report results that are quite impressive at first sight, a closer look usually reveals severe issues with regard to perceptual-motor confounds of the workload manipulations chosen. These confounds render it rather difficult to unequivocally attribute classification accuracies to levels of WML alone. Moreover, as the study of Brouwer et al. (2012) demonstrated, even if motor signals are not confounded with levels of WML, they might nevertheless create high levels of noise in the EEG data that can complicate the effective detection of the more subtle signals resulting from cognitive states. This is particularly the case when time intervals that contain strong motor signals are not excluded from the EEG analysis, which is true for all studies reviewed in this section. However, this type of temporal filtering will probably not suffice to counteract the problem of perceptual-motor artifacts in general. A better option with regard to this issue would probably be the development of more sophisticated methods for the analysis of oscillatory EEG data based on Independent Component Analysis (ICA, Makeig et al., 1996). ICA aims at reconstructing independent sources of neural activity inside the brain from the EEG data observed at surface electrodes. Once ICA-based methods become sufficiently sophisticated to rule out that classifiers strongly rely on the contribution of perceptual and motor sources, confounds like the ones observed in the abovementioned studies might become less severe (see Zander et al., submitted, for an example of how this type of methodological approach might look like). However, until these more advanced methodologies based on ICAs are available for practical application, we would strongly recommend avoiding perceptual-motor confounds when training classifiers for the detection of different levels of WML.

Thus, the rationale that we would advocate with regard to our instructional goal in this paper comprises three parts: First, we would suggest to avoid realistic instructional tasks of different WML for classifier training because these tasks will be necessarily confounded with regard to their perceptual and motor requirements. Second, we would encourage using very well designed tasks from working memory research without perceptual-motor confounds for sound classifier training. And third, in order to render these working-memory tasks relevant with regard to our practical goal, methods of cross-task classification are indicated for the later application of the classifier training to instructional target tasks. However, as the studies reviewed in this section might have sufficiently demonstrated, avoiding perceptual-motor confounds that can be erroneously picked up by classifiers remains a challenge even when using low-level working-memory tasks for classifier training. In the next section we will argue that overcoming this challenge is an important prerequisite for the successful application of cross-task classification methods.

## How to allow for classifier transfer? the case for cross-task classification

The method of cross-task classification for detecting WML is based on the use of EEG data recorded during solving simple and short, but theoretically well-defined working-memory tasks that induce differences in WML without inducing other substantial differences (particularly with regard to perceptual or motor processing). These tasks are used for classifier training. After the levels of WML induced by these tasks have been calibrated, the classifiers then can be applied to target tasks, for instance, more complex instructional tasks. For cross-task classification (other than for within-task classification) all trials of the training data can be used to calibrate the classifier. Subsequently, each trial of the test data serves as new and independent input data to test the separability of the target classes according to the pre-trained classifier model. However, it requires quite sophisticated passive BCI methods to train classifiers on working-memory tasks and to apply these classifiers to more complex learning materials for the detection of different levels of WML by means of cross-task-classification. The main challenges of this approach are (1) identifying or designing appropriate training tasks without confounds for different levels of WML, (2) identifying EEG features related to WML, and (3) developing machine learning algorithms enabling cross-task classification of these features (Schölkopf and Smola, 2002; Lal et al., 2004; Besserve et al., 2007; Brugger et al., 2008). Achieving high classification accuracies requires a good generalizability of the classifier as well as suitable working-memory tasks for classifier training that induce the same type and level of WML than the instructional target tasks.

With regard to our instructional goal, defining a successful classifier for cross-task classification would not only solve the core problem of perceptual-motor confounds when training classifiers with realistic tasks but would also address three other important challenges of detecting levels of WML during complex learning. First, collecting data for classifier training during complex learning tasks is very time consuming for learners, as a sufficiently high number of training trials are needed to calibrate a reliable classifier. Second, realistic learning tasks are not as reproducible as performance tasks because they inherently induce learning effects. Thus, using a set of similar learning tasks of identical complexity does not imply that these tasks will induce the same amount of WML onto learners if administered in a sequence. Rather, the first learning tasks in the sequence might yield much higher levels of workload than the last learning tasks in the sequence due to learners' knowledge gains (cf. the discussion of expertise-reversal effects in Section Cognitive workload and adaptive instruction: A challenge for instructional design). Third, a classifier trained on complex tasks usually remains quite opaque with regard to what characteristics of learners' mental state the classifier actually has picked up. Simple and theoretically well-defined working-memory tasks without perceptual-motor confounds, on the contrary, will be conceptually much more revealing. Thus, an appropriate cross-task classification approach might not only serve to overcome important methodological and practical problems of classifier transfer but might also solve theoretical problems. Up to now, there are only a few studies on applying cross-task classification procedures to workload detection and as can be seen from these studies, defining training tasks for cross-task classification is in no way trivial if perceptual-motor confounds are to be avoided. In the previous section, we already discussed the study by Heger et al. (2010) who used ANNs for cross-task classification but had strong motor confounds in the training tasks (resting state vs. flanker tasks or switching tasks) as well as in in the target tasks (reading vs. typing). Accordingly, the strong cross-task classification results in this study presumably demonstrated the cross-task classification of motor activity vs. no-motor activity but not a cross-task classification of levels of WML.

Gevins et al. (1998) also used ANNs trained on EEG data obtained during solving two types of simple working-memory tasks (verbal and spatial n-back). They were able to successfully discriminate between three levels of WML for each of these tasks. Additionally, they used cross-task classification to distinguish between low and high difficulty levels across the two tasks and reported classification performances above 85%. Although these results are promising at first sight, it has to be noted that subjects basically had to solve the same task (n-back) in a verbal and a spatial version. Using two versions of the same task for cross-task classification is prima facie much simpler than our goal of using rather diverse tasks like a working-memory task and a complex learning task. In our current context, we are obviously more interested in cross-task classification across diverse tasks than in cross-classifying different versions of the same task.

Another study that uses cross-task classification to distinguish different levels of WML across diverse working-memory tasks is the study of Baldwin and Penaranda (2012) that has already been discussed in the previous section. The authors achieved good with-task classification results for three different working-memory tasks. These results might, however, easily go back to some perceptual-motor confounds as described above. For cross-task classification, ANNs were trained on one (or a set of two) of these tasks and tested on a different task, which was not included in the training set. In this analysis, only very poor classification accuracies could be achieved. Actually, according to the authors, the accuracies for each individual subject were near chance level for all possible combinations of tasks used for cross-task classification. From our perspective, these disappointing cross-task classification results could go back to the same perceptual-motor confounds we pointed out earlier as potential explanations of the good within-task classification results: If classifiers can pick up strong perceptual-motor signals that incidentally distinguish between different versions of individual tasks (high vs. low WML), a good accuracy for within-task classification can be expected although this classification does not rely on levels of WML. Unfortunately, if different tasks have different patterns of those distinct perceptual-motor signals, training classifiers on one of these tasks will not yield transfer to the other tasks. Alternatively, the authors themselves assume that the three different tasks they used in their study might induce highly dissimilar features in the EEG signal because they rely on separate types of working memory processes related to different neural structures. In our view, this explanation addresses an important additional issue and strongly points to an urgent need for a better theoretical underpinning of passive BCI approaches to WML with regard to relevant working memory processes, their taxonomy, and their neural basis. In our view, a better theoretical understanding of different working memory processes is not only important for designing suitable sets of training tasks for cross-task classification but also for choosing appropriate strategies for data analysis as will be pointed out in the next section. Thus, for achieving our instructional goal of designing passive BCIs for cognitive load estimation during learning we tried to establish a strong link to state of the art research on working-memory processes.

## How is working memory working? the need for theory on working memory processes

When referring back to the multicomponent working memory model by Baddeley (1986, 2000, 2012), which is the basis of most instructional approaches to cognitive load, sets of tasks like those chosen by Baldwin and Penaranda (2012) indeed seem to be quite disparate with regard to the process components involved. Baddeley's model distinguishes different temporal storage components for handling diverse representational codes as well as a central attentional control system for implementing executive functions. The tasks employed by Baldwin and Penaranda (2012) differed on the one hand in their requirements with regard to processing diverse representational codes like letters, numbers, sentences, and spatial locations. On the other hand, they also differed with regard to the attentional control processes involved like updating and matching a set of items (n-back), interleaving sentence processing with maintaining a list of items (reading span) or maintaining a list of items in the presence of distracting stimuli (Sternberg task). Thus, when considering the different working-memory components postulated by Baddeley, these tasks seem to be quite disparate. Accordingly, a strong cross-task classification performance between them might not be expected to occur. In other words, if three tasks are used for cross-task classification, but each task requires different working-memory processes with regard to storage as well as with regard to attentional control, then no strong classifier transfer between these tasks should be hypothesized unless the different processes involved in the tasks are characterized by an identical or very similar neural basis. Taking this line of reasoning very seriously, we propose that a first step for defining a theoretically sound set of training tasks for cross-task classification should be an exact definition of the cognitive processes that need to be picked up by a classifier. From our perspective, this step is crucial to allow for an appropriate classifier transfer with regard to a later target context. Thus, for our current purposes of connecting working-memory tasks and learning tasks by means of cross-task classification it will be necessary to decide first, which of the different working-memory processes we want to track. Accordingly, we have to clarify which working-memory processes are pivotal when it comes to the role of WML for learning.

Instructional theories like CLT and CTML unequivocally focus almost entirely on processing requirements with regard to the storing of specific representational codes in working memory. Thus, they would consider the capacity limitations of the storage components of working memory to be the main bottlenecks for learning, assuming that these components constrain the amount of new information that can be processed simultaneously in order to be integrated into long-term memory. According to these theories, the entire learning process is hindered when the amount of information a learner has to process exceeds the capacity of these storage components. Using this theoretical account as a basis for an approach to cross-task classification would probably imply to define different classifiers for different storage components in order to measure the WML with regard to one specific storage component. Consequently, each classifier would be trained on a set of working-memory tasks using only one representational code and therefore imposing WML only with regard to one specific storage component. Interestingly, however, recent research on the relation between working memory and instruction suggests a quite different theoretical perspective. Many studies in instructional contexts have shown that the individual capacity of the storage components of working memory (as measured, for instance, by simple span tasks) neither predicts comprehension and learning outcomes in the short run (e.g., in studies on multimedia learning, see Schüler et al., 2011 for an overview) nor in the long run (e.g., in studies on school achievement, cf. Hoard et al., 2008; Alloway and Alloway, 2010; Kornmann et al., submitted). Instead, the central executive functions of working memory (that is, the attentional control processes that are usually required to accomplish more comprehensive working-memory tasks; cf. Daneman and Carpenter, 1980; Engle, 2002) seem to be highly predictive for successful learning and comprehension. These findings render only little support for the basic assumption of current instructional theories that capacity limitations of the storage components of working memory are pivotal for learning and comprehension. Rather, they provide evidence for an alternative idea, namely that the requirements imposed onto attentional control processes in working memory might be the crucial constraints for learners that need to be handled by providing workload-adaptive instructional environments.

Working out this idea in further detail first raises the issue of defining more precisely the nature of attentional control processes in working memory. Baddeley's working-memory theory might be a good conceptual starting point in this respect because it already postulates a central attentional control system for implementing different executive functions. Baddeley has modeled this central executive after the supervisory attentional system postulated by Norman and Shallice (1986). According to Norman and Shallice, many cognitive and overt responses are elicited quite automatically and based on activations of well-learned schemata in long-term memory. However, in order to cope with situations that cannot be handled by schema-based processes alone without resulting in errors, they postulate a supervisory attentional system that actively steps in to inhibit automatic responses and to select more appropriate ones. According to this view, the main role of attention control or executive functions in working memory might be to replace automatically activated information by more suitable information in order to avoid inappropriate processing (for a similar recent account of working memory see Oberauer, 2009). This general idea of attentional control is well in line with a latent variable analysis of executive functions conducted by Miyake et al. (2000). In this study the authors measured individual task performances in a set of simple tasks—each loading on one single executive function—as well as in a set of complex executive tasks. This procedure allows analyzing the contributions of each executive function to the individual performances in the complex task. Confirmatory factor analysis indicated that there are three target executive functions that are clearly separable although they are also correlated with one another. According to this analysis, attention control can be decomposed into the three basic executive functions inhibition, shifting, and updating, all of which aim at replacing currently active working-memory contents. The authors summarize their results by claiming that it is important to recognize both the unity and diversity of executive functions, implying that there is one overarching common factor representing the overall control of attention in addition to three specific factors representing the three executive functions.

Coming back to our instructional context, the ideas of Norman and Shallice (1986) and Miyake et al. (2000) might suggest a distinction between (1) those instructional situations that allow for a schema-based processing and therefore require little attentional control and (2) those situations that highly depend on executive processes (e.g., for selecting relevant information and *inhibiting* irrelevant information, for *updating* and organizing memory contents or for *shifting* between different task demands). Interestingly, and in line with this reasoning, most instructional design principles elaborated by CLT and CTML indeed address issues of helping learners to focus on relevant and to ignore irrelevant information, to update and organize memory contents and to shift attention between different task demands (cf. Mayer, 2009). Therefore, these design principles fit nicely into the view that the amount of controlled attention required by a learning task is pivotal to define relevant WML in instructional contexts. Moreover, a focus on requirements with regard to controlled attention (instead of storage requirements) would also be well aligned to core assumptions of many recent working memory theories (e.g., Barrouillet et al., 2004; Engle and Kane, 2004; Cowan, 2005, 2014; Unsworth and Engle, 2007; Oberauer, 2009). These theories agree that attentional control demands rather than temporary storage demands constitute the core of WML. According to this perspective, the limited ability to control attention constitutes the essence of human working memory limitations and also explains individual differences in working memory capacity (Engle and Kane, 2004; Unsworth and Engle, 2007). Using this theoretical account as a basis for cross-task classification would first of all imply a very different approach to the selection of training tasks compared to the approach of focusing on the limitations of different storage components of working memory. In particular, in order to define a theoretically sound set of training tasks for cross-task classification, we would either need a training set that allows a classifier to pick up the overarching common factor representing the general control of attention or we would have to define three training sets that more specifically address the three executive functions identified by Miyake et al. (2000).

An important consequence of these theoretical insights from the cognitive psychology of working-memory in the context of designing passive BCIs for cognitive load estimation would probably be that we need to better specify the neural basis of the general control of attention in working memory as well as the neural signatures of the three target executive functions that constitute controlled attention. This is important mainly because it may help to inform feature selection for analyzing sets of training tasks for different aspects of WML. Based on functional magnetic resonance imaging (fMRI) techniques, it has been shown that working-memory tasks typically involve an interaction of the dorsolateral prefrontal cortex and the intraparietal sulcus, which are also described as anterior and posterior attentional systems in controlled attention tasks (Curtis and D'Esposito, 2003; Klingberg, 2009). This is well in line with known relations between WML and frontal and parietal changes in oscillatory power (Gevins et al., 1997; Jensen and Tesche, 2002; Stipacek et al., 2003; Missonnier et al., 2006; Krause et al., 2010; Sauseng et al., 2010). It has to be noted, however, that due to volume conduction EEG signals obtained in frontal and parietal electrodes do not necessarily indicate that the sources of these signals also originate from frontal or parietal areas of the cortex. Therefore, fMRI data and EEG data resulting from the same cortical processes do not necessarily need to map with regard to the localization of activated voxels in the cortex and the localization of electrodes on the surface of the skull that are responsive to these processes. Nevertheless, fMRI data are highly useful in our context in order to characterize the homogeneity or heterogeneity of brain areas involved in the different working-memory functions we are interested in. Neuroimaging studies (cf. Nee et al., 2012 for a recent review of 37 studies, Smith and Jonides, 1999; Sylvester et al., 2003) as well as some recent EEG studies (Chapman et al., 2007; Kiss et al., 2007; Hanslmayr et al., 2008; Sörqvist and Sætrevik, 2010; Nigbur et al., 2011) suggest, for example, that beyond the general fronto-parietal pattern of activation characteristic for WML, different frontal areas are involved in different working-memory functions. Detailed fMRI studies show, for instance, that the dorsolateral prefrontal cortex (Broca area 9 and 46) is typically activated when holding spatial information, monitoring and manipulating information in working memory, using strategies to facilitate memory, or verifying representations that have been retrieved from long-term memory (Goldman-Rakic, 1994; Owen, 1997; Dobbins et al., 2002; Bor et al., 2004; for an overview see Owen et al., 2005). Closely related areas in the mid-ventrolateral prefrontal cortex (Broca area 45 and 47) are activated during the selection, comparison and evaluation of stimuli held in short-term and long-term memory as well as during the holding of non-spatial information in working memory and the elaborated encoding of information into episodic memory (Goldman-Rakic, 1994; Petrides, 1994; Henson et al., 1999). Compared to the frontal cortex, the posterior areas of the brain that are crucial for working memory—including the parietal cortex—are mainly responsible for the maintenance of information in working memory. Thus, these areas have also been described as a “buffer for perceptual attributes” (Callicott et al., 1999). In line with this characterization, the involvement of different representational codes in working-memory tasks might lead to subtle differences with regard to the activation of these parietal areas. For instance, Knops et al. (2006) demonstrated in an fMRI study that two types of n-back tasks (identity match: “stimulus is the same/not the same as *n* trials before” and comparison: “stimulus is smaller/larger than *n* trials before”) with numerical vs. verbal materials activated slightly dissimilar areas in the intraparietal sulcus. Besides prefrontal and parietal areas, also the anterior cingulate cortex (ACC) shows an increased activation during working-memory tasks. Activity in this brain region is often related to increased effort, error detection, and attention (Callicott et al., 1999). Moreover, the brain regions crucial for working memory are not operating in isolation from each other, but are communicating. For instance, a study by Honey et al. (2002) has shown that the connectivity of fronto-parietal brain networks increases when working memory is involved.

What are the implications of these findings from the neuroscience literature on working memory and executive functions with regard to potential approaches to the cross-task classification of WML? First, in line with the functional evidence from cognitive psychology (e.g., Miyake et al., 2000) the neural basis of working memory also seems to be characterized by unity (e.g., fronto-parietal network activation) as well as diversity (e.g., differential involvement of specific brain areas). There seem to be networks that are involved in all working-memory tasks whereas other networks are only involved in specific working-memory tasks. Second, we take this pattern as evidence that it might make sense to look out for a quite broad neural signature indicating the load onto a generic common factor representing the overall control of attention (cf. Klingberg, 2009). Third, although there is evidence that particular areas are involved when it comes to specific working-memory functions (e.g., certain executive functions or functions related to particular representational codes), it might be difficult to distinguish these different functions by means of their neural signatures in the EEG signal. Differences in the areas involved are quite subtle so that detecting these specific signatures might require a further development of more sophisticated methods for the analysis of oscillatory EEG data based on ICAs (Makeig et al., 1996), also including the activity of networks (Mullen et al., 2010). These future methods might allow for better defining (source-based) features, which might enable classifiers to pick up these rather subtle differences with regard to the activated brain areas. In line with this evidence, in our own attempts to classify EEG data from working-memory tasks, we will first try to identify an overarching factor of controlled attention involved in WML. This factor is assumed to represent the unity of executive functions according to the analysis of Miyake et al. (2000) and is considered to be based on the same neural basis (i.e., fronto-parietal networks, Klingberg, 2009) for different learning materials, independent, for instance, from the representational codes involved (e.g., numerical or verbal) or from the specific executive functions required (e.g., inhibition, shifting, updating). After having specified more precisely the nature of the working-memory processes we want our classifier to pick up—based on the cognitive as well as on the neuroscientific working-memory literature—we can now continue to develop a theoretically sound set of training tasks for these processes.

Defining a classifier (based on fronto-parietal patterns of activation in EEG data) that allows extracting the amount of controlled attention required by a task (independent from specific representational codes or executive functions involved) presupposes at least a set of two different working-memory tasks for classifier training, each without perceptual-motor confounds, and both differing substantially with regard to the representational codes and executive functions they involve (to ensure that the classifier will not extract these specific features). An ideal set of working memory-tasks for classifier training in our instructional setting would consist of two working-memory tasks that are both known to be highly correlated with achievements in learning tasks (potentially indicating that the executive functions involved in these tasks are relevant for learning). At the same time they should not be highly correlated with each other (potentially indicating that these tasks do not involve the same combination of executive functions). Actually, a combination of an n-back task with a complex span task—as used in the study of Baldwin and Penaranda (2012)—fulfills these constraints. Both tasks are standard paradigms in working-memory research that predict learning outcomes very well (Daneman and Carpenter, 1980; Engle, 2002; Kornmann et al., submitted) but correlate only weakly with each other (Kane et al., 2007; Redick and Lindsey, 2013), indicating that different executive processes might be involved in task performance. For instance, a reading span task requires *shifting* between a rather complex semantic processing task and a rather simple additive *updating* of a set of items. Contrarily, the n-back task requires a complex *updating* of a set of items involving a replacement (*inhibition*) of previous set members and a reordering of the sequence of set members as well as a *shifting* between these updating task demands and a simple identity-matching task. In the light of this analysis it is quite astonishing that the promising combination of an n-back task and a complex span tasks in the study of Baldwin and Penaranda (2012) yielded no positive cross-task classification result with regard to the Sternberg task. One reason for this finding might be of course that there were differential perceptual-motor confounds of the three tasks in that study (as already mentioned above). Another reason, however, might be that the Sternberg task used in the study does not really represent a working-memory task in the sense that it involves executive demands like *updating, shifting*, or *inhibition* (in addition to maintaining a set of items): Subjects had to maintain a sequentially presented list of items for 2 s and after this phase they had to decide whether a certain probe was present in the set or not. Subsequently, a new list was presented. Thus, this task does not involve maintaining a memory set and working on this set or on other items in an interleaved fashion. Therefore, the Sternberg task resembles much more a short-term memory task than a working-memory task. In line with this reasoning, Corbin and Marquer (2013) argue that the Sternberg task can be considered a working-memory task only under very specific conditions, for instance, when additional experimental constraints are imposed on subjects that increase the processing load. Thus, in terms of neural signatures, the Sternberg task may mainly rely on neural networks not involved in executive control whereas the n-back task and the complex span task may rely on neural networks required for executive control, but may differ with regard to the specific mixture of executive functions required for task performance, which explains their rather weak correlation on a behavioral level. In essence, as our overview of the working-memory literature on the behavioral and the neural level reveals, it will not be sufficient to just select a random working-memory task or a random combination of working-memory tasks for successful classifier training when cross-task classification is intended. Rather, a strong link to state of the art research in the cognitive psychology and cognitive neuroscience of working-memory processes is necessary to select appropriate sets of working-memory tasks that are characterized by overlapping task demands with regard to the target construct (controlled attention) and differential mixtures of specific executive functions so that the resulting classifier is not constraint to a specific mixture of these functions.

## What have we learned so far? a summary in five lessons

As a summary on how the different issues raised in this paper might be overcome in order to achieve the instructional goal of designing passive BCIs for cognitive load estimation during learning we compiled the following list of five lessons learned. These lessons do not only result from research on cognitive load in instructional design (Section Cognitive workload and adaptive instruction: A challenge for instructional design) and from studies on EEG measures of workload during instruction (Section Promises and drawbacks of EEG-based measurement of WML during learning: The issue of perceptual-motor confounds), but also from our own initial studies (Section Study 1: Realistic instructional materials imposing different levels of WML: Do single-subject single-trial EEG data allow for a classification? And what is being classified?), from studies on workload classification (Section Are there successful classifications of levels of WML based on oscillatory EEG data? A critical reflection on recent studies and some suggestions for improvement) and on cross-task classification (Section How to allow for classifier transfer? The case for cross-task classification) as well as from the behavioral and neurocognitive working-memory literature (Section How is working memory working? The need for theory on working memory processes). It should become clear from this list that many details of how to choose tasks for classifier training and how to analyze the EEG data resulting from these tasks might be of crucial importance, even though these details might appear quite oversophisticated at first sight. In order to demonstrate the importance of these lessons we applied them meticulously in designing a follow-up study (Walter et al., submitted) that resulted in a successful cross-task-classification of WML. This study will be summarized in the next section.

### Lesson 1 – the role of task order in the context of learning

A randomized task order is usually a good choice for classifier calibration and testing to avoid any confounds of the classes to be detected with the time of presentation. however, when it comes to learning tasks, randomization is hard to implement. wml is a highly volatile learner characteristic that changes during learning not only due to ubiquitous increases in the complexity of instructional materials and task requirements over time (objective complexity) but also due to learners' increasing levels of knowledge (degree of expertise, cf. section cognitive workload and adaptive instruction: a challenge for instructional design). based on these changes, task order plays a different role for learning tasks than for performance tasks where a randomized task order can typically be implemented without hesitation. for learning tasks, randomization is usually not appropriate. in most cases, certain materials will be too complex to be understood at an early phase of instruction while they might be quite easy at a later point in time. this is due to the fact that an increase in expertise over time (i.e., learning) allows handling more complex contents than before without the need to activate a larger number of knowledge structures in working memory (due to the chunking of information into more complex concepts, cf. section cognitive workload and adaptive instruction: a challenge for instructional design). accordingly, two materials with identical objective complexity might impose very different levels of wml at different points in time during learning due to learners' knowledge gains. as a consequence, it is not advisable to present too complex learning tasks at the beginning of an instructional sequence and too simple learning tasks at the end of an instructional sequence. otherwise, no learning might occur at all because the learning tasks are either much too simple or much too complex in relation to learners' current knowledge prerequisites. this inherent dynamic of wml in the context of learning tasks due to the ongoing acquisition of knowledge implies that it makes no sense to present learning tasks in a randomized task order, as one would typically do with performance tasks: realistic learning tasks differ exactly from performance tasks with regard to the fact that they cannot be presented in an arbitrary order without jeopardizing their character as learning tasks. this specific characteristic, however, has serious implications for classifier testing and training when designing passive bcis for cognitive load estimation during learning: most important, when instructional materials need to be presented in a fixed simple to complex sequence in any realistic learning context, it is inevitable that testing a classifier on such a fixed sequence of learning materials is subject to a confound of objective levels of task complexity and presentation time. from our perspective, it is necessary to take the potentially negative effects of such a confound due to fixed task order explicitly into account (e.g., the effect that a classifier might pick up slow drifts in the eeg signal over time as useful information about task complexity due to the correlation of both factors). we advocate two measures to counteract these potentially negative effects. first, with regard to feature selection for classifier definition, we suggest to use %erd/ers ratios (Pfurtscheller and lopes da silva, 2005) instead of simple power values to explicitly cancel out slow changes in the eeg signal over time. the %erd/ers ratios are calculated by using a baseline signal for comparison that immediately follows or precedes the target window of analysis. second, for parity reasons we recommend to use the same simple to complex sequence not only for classifier testing on learning tasks but also for classifier training on working-memory tasks (by presenting the working-memory tasks needed to train the classifier in a similar fixed order. when applying %erd/ers ratios to both task sequences (classifier training and testing), the baseline as well as the window of analysis will both be subject to potential drifts in the eeg signal over time for all sequences. accordingly, these drifts cannot be erroneously picked up by a classifier.

### Lesson 2 – classes based on subjective ratings of WML

The inherent dynamic of wml due to learning explained in lesson 1 also has a second implication beyond task order, namely that it might not be useful to define classes of learning tasks for classifier training and testing based on their objective task complexity. rather, the definition of these classes needs to take into account that the same learning tasks could elicit different levels of wml depending on learners' current degree of expertise as well as on their individual working-memory capacity (cf. section cognitive workload and adaptive instruction: a challenge for instructional design). for instance, in a block of 40 learning tasks of similar objective complexity, the first 20 tasks might impose rather high levels of wml onto learners due to their novelty, whereas the last 20 tasks might impose lower levels of wml onto learners due to schema formation (chunking). accordingly, in line with instructional theories (cf. section cognitive workload and adaptive instruction: a challenge for instructional design) we propose that classes based on subjective ratings of wml may be more appropriate for classifier training and classifier testing than classes based on objective task complexity, at least when the target construct for classification is the wml actually experienced by learners (which is the case in the context of adaptive instructional environments). for illustration, consider the example of learning how to solve two-digit addition tasks in the octal numeral system (base-8 number system, e.g., 23 + 77 = 122). this task will be quite demanding with regard to working-memory resources when encountered for the first times. however, with increasing practice, it will become rather easy, comparable to two-digit addition tasks in the decimal numeral system that we are all acquainted with (e.g., 23 + 77 = 100). thus, the wml actually experienced by learners when solving two-digit addition tasks in the octal numeral system will change to a large extent over time, whereas the objective task complexity remains constant. this example should make clear that the classes that we need to detect in instructional contexts are subjective levels of wml and not objective levels of task complexity.

### Lesson 3 – avoiding perceptual-motor confounds

The windows used for analyzing eeg data during classifier training should not contain perceptual-motor confounds that could be erroneously picked up by the classifier. this implies that classifier training cannot be conducted on realistic learning tasks but need to be restricted to more controlled experimental working-memory tasks. additionally, methods like ica and connectivity analyses need to be further developed to better rule out motor artifacts in the future (cf. section promises and drawbacks of eeg-based measurement of wml during learning and instruction: the issue of perceptual-motor confounds, study 1: realistic instructional materials imposing different levels of wml: do single-subject single-trial eeg data allow for a classification of these levels?, are there successful classifications of levels of wml based on oscillatory eeg data? a critical reflection on recent studies and some suggestions for improvement, how to allow for classifier transfer? the case for cross-task classification). these methods might eventually allow excluding those components or networks from the set of features for classifier training, which clearly reflect perceptual or motor processing.

### Lesson 4 – combining working-memory tasks

The working memory-tasks used for classifier training should not be confined to a single type of task. instead combinations of at least two working-memory tasks should be used (preventing the classifier from picking up task-specific features). all working-memory tasks used should be predictive for achievements in learning tasks (potentially indicating that the executive functions involved in these tasks are relevant for learning). at the same time, the tasks should differ with regard to the specific executive functions they require (updating, shifting, inhibition) and with regard to the representational codes (numbers, letters, words etc.) they involve in order to train a generic classifier sensible to general changes in the requirements on controlled attention irrespective of specific executive functions or representational codes. this can be ensured by selecting working-memory tasks that only have a weak correlation with each other (potentially indicating that these tasks do not involve the same combination of executive functions). additionally, it might be wise to avoid visual-spatial working-memory tasks for classifier training because these tasks might afford rehearsal strategies that produces different pattern of eye-movements for different levels of wml (cf. section how is working memory working? the need for theory on working memory processes).

### Lesson 5 – difficulty level of working-memory tasks

One should be prepared that the level of wml induced by controlled experimental working-memory tasks used for classifier training could differ from the level of wml induced by realistic learning tasks used for classifier testing. this is a natural implication of the fact that different task are used for classifier training than for classifier testing (cf. section how to allow for classifier transfer? the case for cross-task classification). thus, a calibration procedure for cross-task classification might be needed that allows for a scaling to adjust a trained classifier to new testing materials.

## Study 2: cross-task classification of basic working-memory tasks and realistic instructional tasks

Based on the five lessons outlined in the previous section, we extended the design of our Study 1 (outlined in Section Study 1: Realistic instructional materials imposing different levels of WML: Do single-subject single-trial EEG data allow for a classification of these levels?, for details see Walter et al., 2011) in several ways (Walter et al., 2013a; Walter et al., submitted). The goal of Study 2 was to develop efficient classification methods to differentiate levels of WML based on cross-task classification. We trained a SVM on two well-controlled working-memory tasks (reading span, numerical n-back), assuming theoretically that they would induce similar types of WML (and accordingly similar types of neural processing) as the two realistic learning tasks we used subsequently for classifier testing (algebra problems). By taking into account the lessons summarized in the previous section, we were able to achieve promising results for cross-tasks classification. 21 subjects participated in the study, however, due to technical problems during data collection, five subjects had to be discarded from the analysis. For the majority of the remaining 16 subjects the cross-task classification reached a significant classifier performance (*p* < 0.05, permutation test), with classifier accuracies up to 95% for the best subjects. The classifier was able to distinguish the subjectively easier vs. harder set of algebra problems with a mean classification accuracy of 73%. In the following we will outline the methodological strategies that we used to overcome the inherent challenges of using cross-task classification for cognitive workload assessment in the context of learning.

### Task design

#### Training tasks

According to the considerations in Section How is working memory working? The need for theory on working memory processes, it will not be sufficient to just select a random working-memory task or a random combination of working-memory tasks to train an efficient classifier for cross-task classification. Rather, it will be necessary to select appropriate sets of working-memory tasks overlapping in task demands with regard to our target construct (controlled attention demands due to WML) and differing with regard to specific executive functions (see Lesson 4). As described above, the n-back task in combination with the reading-span task fulfills these requirements. Thus, we decided to use this combination of tasks in Study 2. Both tasks predict achievements in learning tasks very well but correlate only weakly with each other, which might be due to their different profiles with regard to executive functions. A reading span task requires *shifting* between a semantic processing task and a simple additive *updating* of a set of items. The n-back task, on the contrary, requires an *updating* of a set of items together with a replacement (*inhibition*) of previous set members interleaved with an identity-matching task. In addition to these differences, we designed the tasks to cover various representational codes (see Lesson 4). In the n-back task, single-digit numbers had to be memorized (except the number seven, which is the only two-syllable number), whereas the reading span task was based on memorizing letters (from the set B, F, H, J, L, M, Q, R, X) and on verifying sentences. By these design decisions we tried to prevent the classifier from picking up task-specific features. We implemented three levels of task difficulty for each task in a within-subject block design (i.e., 1-back, 2-back, 3-back and readings spans with 2, 4, 6 letters/sentences). We ensured that subjects received identical visual displays in all levels of task difficulty to avoid perceptual confounds (see Lesson 3).

#### Testing tasks

For classifier testing, two different types of algebra word problems were designed (subtraction problems and fraction problems). For both types of tasks, again three levels of task difficulty were implemented that strongly differed with regard to the level of WML they would induce. To avoid perceptual confounds of task difficulty (see Lesson 3), all word problems contained exactly four numerical pieces of information at each level of task difficulty (either numbers or fractions). Additionally, the word problems were matched for number of words. Thus, we ruled out that more difficult word problems resulted in more numerical of text information or more complex visual displays leading to differences in processing, which may show up in the EEG (see Section Are there successful classifications of levels of WML based on oscillatory EEG data? A critical reflection on recent studies and some suggestions for improvement).

In order to solve the subtraction problems, a variable *x* had to be calculated by selecting and integrating appropriate numbers. For the first level of task difficulty, subjects merely had to select one out of the four numbers (*x* = *a*). The second level required them to subtract two relevant numbers (*x* = *a* − *b*). The third level, finally, asked for the difference between two differences, thus involving all four given numbers (*x* = (*a* − *b*) − (*c* − *d*)). This manipulation of task difficult was based on the taxonomy by Schnotz et al. (2010) and can be assumed to induce strong differences in WML.

The fraction problems required subjects to select and instantiate algebraic expressions containing fractions and multiplications in order to determine the value of a variable *x*. For the first level of task difficulty, the appropriate expression contained one fraction (*x* = *c* · *a*/*b*). For the second difficulty level the expression contained the same fraction two times (*x* = *c* · *a*/*b* + *d* · *a*/*b*). The third level contained two different fractions (*x* = *c* · *a*/*b* + *d* · *e*/*f*). This manipulation of task difficult was based on the taxonomy by Scheiter et al. (2010) and can be assumed to induce strong differences in WML.

In all four tasks, subjects had to react with key presses to provide their answers. They could react either with “yes” or “no” in the working-memory tasks (identity matching in the n-back task and sentence verification in the reading-span task) or with one out of four multiple choice options in the learning tasks used for classifier testing). The motor reaction was exactly the same for both working-memory tasks and both learning tasks. Furthermore, there were no differences in the motor reaction between different levels of tasks difficulty. No feedback was given to subjects in order to avoid confounding task difficulty and ratio of negative feedback (cf. Section Are there successful classifications of levels of WML based on oscillatory EEG data? A critical reflection on recent studies and some suggestions for improvement).

For modeling a realistic learning scenario (cf. Lesson 1), the mathematical word problems were presented in a simple to complex sequence for each learner (within-subject block design). Accordingly, a randomized task order for classifier testing could not be implemented. For parity reasons, we used the same simple to complex sequence for each working-memory task to generate the data used for classifier training. To rule out potential negative effects of such a fixed task order (e.g., slow drifts in the EEG signal that can inform the classifier) we calculated %ERD/ERS ratios using a baseline for comparison that immediately follows or precedes the window of analysis, thus counteracting the potential confound of time and level of WML (for details see below).

### Details of EEG measurement

With regard to the windows used for analyzing EEG data, we ensured that they did not contain any motor events or perceptual confounds that could be picked up by a classifier. The windows of analysis always ended at least 125 ms before any keypress to exclude EEG signals based on motor planning (Grabner and De Smedt, 2011). Furthermore, a time interval of 125 ms after any keypress was excluded from the analysis to avoid potential motor artifacts (see Lesson 3). Although these decisions with regard to the EEG analysis as well as with regard to the tasks designs already yielded highly controlled tasks without perceptual-motor confounds, we additionally reduced eye-movement artifacts by optimizing the data pre-processing step (Walter et al., submitted). We applied the artifact-reduction method described by Schlögl et al. (2007).

To counteract the potential confound of time and level of task difficulty, we calculated %ERD/ERS ratios based on power values using two time intervals for each task, one interval that strongly imposes the task-specific WML (activation interval, *I_a_*) and one interval that imposes only a low level of WML (resting interval, *I_r_*). Defining these intervals requires a theoretically based understanding of the working-memory processes involved in the tasks (cf. Section How is working memory working? The need for theory on working memory processes). We ensured that each trial of a task included both intervals to avoid systematic evects of drifts in the EEG signal (see Lesson 1).

In the n-back task, every 2000 ms a new digit was presented for identity matching. Subjects could react by pressing “yes” or “no” anytime. For *I_a_* we used the time interval from stimulus onset until 125 ms before keypress (in this interval identity matching and updating/replacement is required). For *I_r_* we used the time interval from 125 ms after keypress until the next stimulus appeared (in this interval mainly storage is required).

In the reading-span task, a list of sentences was presented for verification (e.g., oranges are blue). After each sentence, subjects could react by pressing “yes” or “no” anytime. After keypress a fixation cross was presented for 500 ms before a letter (that had to be remembered for later recollection) was presented for 1000 ms. For *I_a_* we used the time interval in which the letter was presented (in this interval a shifting between the semantic processing task and an updating of the set of letters to be remembered is required). For *I_r_* we used the time interval from 125 ms after keypress (sentence verification) until the next letter to remember appears (in this interval mainly storage is required).

In both learning tasks, a series of word problems was presented to subjects. First, subjects read a page with facts as long as they wanted. After keypress a problem statement appeared that had to be solved. Subjects could react by pressing a next-button anytime and could then select one out of four multiple-choice options to provide their solution. Subsequently, a fixation cross was presented for 500 ms. For *I_a_* we used the time interval from the onset of the problem statement until 125 ms before keypress for selecting the problem solution (in this interval the necessary facts had to be remembered and inferences had to be drawn for problem solution). For *I_r_* we used the time interval from 125 ms after keypress (i.e., after providing the problem solution) until the next page of facts appeared (in this interval no cognitive processing was required).

### Data analysis and results

Concerning the feature selection, we focused on frontal and parietal electrodes with regard to the spectral power within the theta (4–7 Hz) and alpha (8–13 Hz) frequency band (see Section Promises and drawbacks of EEG-based measurement of WML during learning: The issue of perceptual-motor confounds and Study 1: Realistic instructional materials imposing different levels of WML: Do single-subject single-trial EEG data allow for a classification of these levels?). For spectral analysis, an autoregressive model was calculated with the Burg-Algorithm. The %ERD /ERS values for the 10 postulated frequencies (4, 5, 6, 7, 8, 9, 10, 11, 12, and 13 Hz) were calculated with regard to 10 electrodes (F3, Fz, F4, FC1, FC2, CP1, CP2, P3, Pz, P4) resulting in 10 features for each of the 10 electrodes. For the sake of consistency, these features were used across all subjects and tasks.

Cognitive-load ratings were obtained after each block of working-memory tasks and after each learning task by means of a 7-point Likert scale. These ratings were used for defining task classes (according to subjective levels of WML, see Lesson 3) and for adjusting the complexity levels of the different tasks (see Lesson 5). Since the tasks (n-back task, reading-span task, and subsequent learning tasks used for classifier testing) differed substantially with regard to the subjective level of WML they induced for each of their three difficulty levels, a calibration procedure was applied (see Lesson 5). We selected two out of the three difficulty levels for each task based on subjects' error rates and cognitive-load ratings. For instance, we removed the first level of task difficulty of the subtraction problems from the analysis because it turned out that these tasks were much simpler than the other tasks. The procedure of selecting two out of three difficulty levels for analysis allows us to calibrate difficulty levels across different tasks. For the remaining differences in the difficulty levels across the four tasks, the scaling method of the EEG power values was improved by scaling the data over trials and by adjusting the range of the diverse datasets: First, our training data were z-score normalized resulting in a centered, scaled version of the input-data. Subsequently, we calculated the means and standard deviations of the power values of the training-set. Finally, the testing data were normalized with regard to these means and standard deviations calculated from the training data.

In order to define classes for the training and testing of classifier we analyzed the relation between the objective complexity levels of the four tasks and the subjective cognitive-load ratings. In line with our expectations (see Lesson 1) and with instructional theory (see Section Cognitive workload and adaptive instruction: A challenge for instructional design), it turned out that due to learning the levels of subjectively experienced cognitive load decreased over time within each level of objective task difficulty. As a consequence, we used subjective cognitive-load ratings for defining classes. For this purpose, we calculated the mean value of the subjectively experienced WML for all working-memory tasks solved by a subject as a personal cut-off point to define two classes (low vs. high level of WML according to cognitive-load ratings) for all four tasks (i.e., we defined the two classes for both types of word problems according to the same subjective cut-off point). Using this procedure requires to adjust the objective complexity levels of the tasks for classifier training and testing according to the calibration procedure described in the last paragraph to ensure that the number of trials in both classes is sufficiently balanced for each individual task (i.e., it is necessary to select appropriate complexity levels of all tasks so that all tasks have a similar range of WML as measured by the subjective cognitive-load ratings).

For cross-task classification, we used a SVM with RBF-Kernel that was trained on the combination of the two working-memory tasks and applied to the two realistic learning tasks. As a result, the two levels of WML of the learning tasks could be successfully classified on a single-subject single-trial basis. On average a classification accuracy of 73% could be achieved for the 16 subjects. The classification results were significant for 22 out of the 32 classifications conducted (for each of the 16 subjects two classifications were conducted for the two learning tasks). It has to be noted, though, that these results are based on classification decisions after each trial of the learning tasks. Taking the results of Brouwer et al. (2012) into account, much higher accuracies could be expected when classification decisions would be based on more than one trial of a task.

In conclusion, the methodological strategies that we used to implement the lessons learned so far yielded quite promising results. Our decisions with regard to how to design and analyze Study 2 allowed for a successful cross-task classification of WML when solving complex learning tasks. Thus, the most important precondition for the goal of developing workload-adaptive instructional environments based on passive BCIs, namely the availability of a continuous and non-intrusive assessment of WML during solving realistic learning tasks, seems to be satisfied.

## Discussion

In this paper, we discussed and applied several lessons learned on how to implement passive BCI methods to assess WML during learning. A study based on these lessons (Study 2) yielded very promising results for a cross-task classification of WML when solving complex learning tasks (see Section Study 2: Cross-task classification of basic working-memory tasks and realistic instructional tasks). In the remainder of this paper we will discuss the prospects for further improving cross-task classification results and for applying our methods outside the lab in realistic environments, for instance by expanding the feature space used for workload assessment in real-world studies.

### Future prospects for improving cross-task classification results

To improve cross-task classification accuracies even further, a couple of additional methods could be applied to the tasks we used in our studies. One issue that might be addressed by these methods is the fact that, due to learners' knowledge gains over time, the learning tasks needed to be presented in a fixed simple to complex order, which results in a confound of presentation time and objective levels of task complexity. Due to non-stationarities in EEG signals over time, a classifier might pick up slow drifts in the feature space as useful information about task complexity due to the correlation of both factors. Although we tried to diminish this effect by analyzing %ERS/ERD ratios, in future studies, additional methods for covariate shift adaptation might help to further alleviate these drifts and to improve classification accuracies (Satti et al., 2010; Spüler et al., 2012b). Another approach would be to present all tasks in a randomized order of difficulty levels to evaluate the influence of non-stationarities on the cross-task classification results. It should be clear from Lesson 1, however, that this manipulation is not very plausible as a modeling of a realistic learning situation (which is our target scenario) but should only be used to clarify the role of non-stationarities for methodological reasons.

Another interesting option that arises from the task design of Study 2 is to explicitly include very high levels of WML in the analysis that intentionally result in a “cognitive overload.” Chanel et al. (2008) found that an excessive increase in WML might lead to disengagement that is detectable by a reversed EEG pattern (i.e., theta-desynchronization and alpha-synchronization), which corresponds to the neural signature of low WML. In our own study we found that absolute difficulty levels varied substantially across different types of tasks. We used the calibration procedure described above to remove difficulty levels, which turned out to be too easy (e.g., arithmetic level 1) or too difficult (e.g., algebra level 3) for learners in terms of error rates and subjective ratings of cognitive load. Interestingly, however, exploratory analyses of these excluded conditions indeed revealed in line with Chanel et al. (2008) the similarity of the neural signatures of conditions with very low levels of WML and very high levels of WML (in the sense of cognitive overload), thereby potentially indicating task disengagement (Walter et al., 2013b). This pattern might be exploited in future studies to better define a zone of optimal workload during learning (high but not too high) and to assess this zone by applying passive BCI methods. Cross-task classification might benefit from this approach because tasks that induce cognitive overload will be better excluded from the high WML class.

### Assessing workload outside the lab in real-world environments

Although we applied our approach of assessing different levels of WML to realistic instructional materials (algebra word problems), the laboratory tasks used for data collection were nevertheless highly controlled with regard to temporal parameters and perceptual-motor confounds. However, if we consider our target scenario of applying passive BCIs in realistic learning scenarios the question remains, whether the methods we developed can be applied successfully outside the lab in real-world environments, where several parameters are expected to differ from the lab. Of course, it is highly interesting in the first step to evoke WML by using well-controlled laboratory tasks as laboratory tasks and designs allow for high control over most of the relevant factors and provide a clear structure that can be aligned to cognitive theories of WML. On the other hand, using well-controlled laboratory tasks moves the whole study further away from realistic settings. It is likely, that the brain works differently in real world scenarios, where tasks appear to be more relevant for the person involved. McDowell et al. (2013) discuss this hypothesis postulating that the activity of the brain increases with the relevance of the tasks it is involved in. Hence, it is of great importance that theoretical and methodological approaches developed in laboratory studies are validated in real world environments. Fortunately, first steps into this direction (e.g., Zander et al., submitted) provide evidence that today's state of the art theories explain at least partially what brain activity is related to realistic workload in real-world environments.

One of the most important issues with regard to real-world studies will be the occurrence of perceptual-motor confounds that have been extensively discussed in this paper. In realistic environments we would clearly expect workload-related behavioral activity, such as specific changes in the pattern of behavior. Due to volume conduction such behavioral activity will be added to the EEG signal through muscularly, ocularly, or translatorly induced effects. Generally, these changes in the potential recorded at EEG electrodes will be bigger in amplitude than those resulting from cortical activity. Hence, it is likely that BCI classifiers might strongly rely on this behavioral information. Thus, in uncontrolled realistic application scenarios it might be quite unclear whether a single-trial detection system used for classification is based on brain activity or on perceptual-motor confounds. This mirrors the methodological problems of many of the studies discussed in this paper.

As a consequence, it is important to better investigate, which signals a BCI classifier picks up when moving out of the controlled laboratory environments. As the EEG is a complex mixture of different signals, complex methodologies have to be applied to identify sources contributing to it. Recent research revealed that methods like ICA (Makeig et al., 1996) are powerful tools for solving this problem. Independent sources of signals can be identified by their temporal, spectral, and spatial properties. From this information the type of process might be inferred that underlies a specific aspect of the overall signal. For instance, data resulting from artifacts might be better discriminated from cortical activity, and cortical activity might be divided into different processes that can be related to specific cognitive and affective states. ICA can even be used to reveal the connectivity of different cortical structures. Such network activity likely carries relevant information about workload, which is not only based on the activation of specific brain areas but also on the information transfer between different areas (e.g., the fronto-parietal networks implementing controlled attention). Different statistical approaches might be utilized in the future to investigate network activity in the EEG. A description of an open-source toolbox including the most commonly used methods can be found in Mullen et al. (2010).

To conclude, we consider passive BCIs to be powerful tools for improving adaptive learning environments in the future, provided that they are properly validated, first, by combining controlled experimental studies and complex real-world studies and, second, by investigating independent components, network activities and classification properties. Moreover, our passive BCI approach might also be transferred to many other aspects of Human-Computer Interaction, using cognitive state monitoring for introducing fundamentally new types of input, like implicit interaction (see Zander et al., 2014).

### Conflict of interest statement

The authors declare that the research was conducted in the absence of any commercial or financial relationships that could be construed as a potential conflict of interest.
